# Relationships between Inflammatory Biomarkers and Fatigue among Patients with Moderate and Severe COVID-19

**DOI:** 10.1155/2023/7057458

**Published:** 2023-04-30

**Authors:** Besher A. Gharaibeh, Jehad Rababah, Obieda Haneyah

**Affiliations:** Jordan University of Science and Technology, Faculty of Nursing, Irbid, Jordan

## Abstract

**Background:**

Patients with moderate or severe COVID-19 infection suffer from varying levels of fatigue; however, there is a lack of understanding regarding the effect of inflammation on fatigue; and whether these relationships differ according to the severity of the infection.

**Aim:**

To assess the relationships between selected inflammatory biomarkers and fatigue levels among hospitalized Jordanian patients with moderate or severe COVID-19 infection.

**Methods:**

A quantitative cross-sectional design was used. A total of 352 participants were recruited for the study. Data regarding fatigue type and level were collected using the Chalder fatigue scale. Laboratory test results regarding several selected inflammatory biomarkers (e.g., ESR, CRP, IL-6, D-dimer, and others) were collected from patient records. The severity of the COVID-19 infection was determined using the criteria of the Ministry of Health in Jordan based on the results of O_2_% (oxygen saturation).

**Results:**

The mean scores of the total fatigue level significantly differed between the two levels of the severity of COVID-19 infection (moderate and severe levels) (*t* = −3.0, *p* < 0.05). Similar findings were observed with physiological fatigue (*t* = −3.50, *p* < 0.05), and no significant difference was observed in psychological fatigue. Out of the selected inflammatory markers, only neutrophil and lymphocyte count had a significant influence on total fatigue level.

**Conclusion:**

The level and type of fatigue was affected by the severity of the disease. However, the disease process itself represented by the levels of the inflammatory markers showed little influence on fatigue. The implications such as continuous screening of fatigue, and monitoring of the levels of the inflammatory markers are important to assist in diagnosing and managing COVID-19 patients. Furthermore, the relationship between the inflammatory process and fatigue is complex and requires further exploration.

## 1. Introduction

COVID-19 is a viral infection that affects the respiratory system, with symptoms that include tiredness and fatigue. The World Health Organization (WHO, 2023) in its report that was updated on 23 December, 2022, indicated that the total number of cases had reached 651,918,402 cumulative cases, with a cumulative death count that reached 6,656,601 deaths. Each day, a total of 778,897 cases are discovered worldwide. In the United States, the Center for Disease Control and Prevention [1] reported in its updated report on 30 December, 2022, that the total cumulative number of cases had reached 100,662,056, and the total number of cumulative deaths reached 1, 088,481. The current reported case fatality rate (CFR) is about 1%. Each day in the U.S. approximately 5668 cases are hospitalized due to COVID-19 infection. The Jordanian Ministry of Health [2] reported as of August 2022, that the total number of cases had reached 1,731,549 and the total number of deaths reached 14105. A total of 159 cases were admitted on the week of the report (13–19/August, 2022), a total of 4832 new cases were reported, and a total CFR of 0.09 per 100,000 of the overall population was observed.

Dhochak et al. [3] describe the pathophysiological process of COVID-19 infection. They stated that the SARS-CoV-2 virus attacks the respiratory epithelial cells by attaching to the angiotensin-converting enzyme-2 (ACE-2) protein using its S-protein. This infection causes the activation of an inflammatory response that includes various mediators which also activates the hemostatic system due to the endothelial dysfunction. In other words, thromboinflammation and cytokine storm play a significant role in the disease process and severity.

The Jordanian Ministry of Health [4] classified COVID-19 in terms of its severity of illness into four categories: mild, moderate, severe, and critical. These clinical forms which were defined in this study according to the criteria of the Jordanian Ministry of Health are as follows.

### 1.1. Mild Clinical Form

It is a confirmed case in which the patient suffers from symptoms and signs of upper respiratory tract infections and does not complain of symptoms of shortness of breath, and the percentage of hemoglobin saturated with oxygen is more than 94%.

### 1.2. Moderate Clinical Form

The patient suffers from symptoms and signs of lower respiratory tract infections (bronchitis or pneumonia), including shortness of breath, and his hemoglobin saturation with oxygen is more than 94%.

### 1.3. Severe Clinical Form

**Figure 1:**
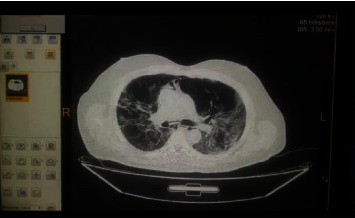
Lung CT scan for a patient with confirmed COVID-19 infection.

**Figure 2:**
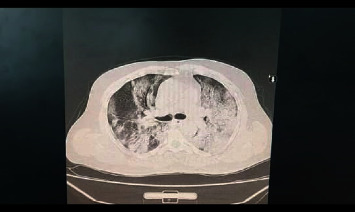
Lung CT scan for a patient with confirmed COVID-19 infection.

**Figure 3:**
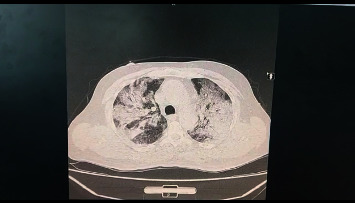
Lung CT scan for a patient with confirmed COVID-19 infection.

The patient who suffers from pulmonary infections with shortness of breath and whose hemoglobin saturation with oxygen is less than 94% (See Figures 1–3 for 3 for computed tomography (CT) scans for the lungs of patients with severe COVID-19 infection).

### 1.4. Critical Clinical Form

COVID-19 is a condition where a person suffers from severe respiratory depression and requires effective artificial respiration, and fatigue is one of the main and commonly reported symptoms of COVID-19, which is considered a subjective sensation of psychological or physical exhaustion that reduces the capacity to perform a psychological or physical task because of the depleted resources [5]. Fatigue is a complex phenomenon that is usually associated with COVID-19 infection and cannot be completely explained by a single mechanism. However, fatigue can be attributed to the underlying inflammatory condition, which is a common thread of COVID-19 infection [6].

Several inflammatory markers have been examined in terms of their relationship with the process of COVID-19 infection. These markers included ESR, IL-6, D-dimer, PCT, and CRP [7]. Certain inflammatory markers such as ESR, PCT, CRP, and other markers were found to positively correlate with the severity of COVID-19; and CRP was described as a sensitive systemic marker of acute-phase response during inflammation and tissue damage, which could be used as an indicator of COVID-19 progress [8].

There is a lack of studies that addressed fatigue during COVID-19 infection in relation to the levels of the inflammatory markers. So, this study aims to describe the relationship between selected inflammatory biomarkers and fatigue among hospitalized patients with moderate or severe COVID-19 infection.

The following are the research questions:

1. What is the relationship between COVID-19 severity and fatigue among hospitalized patients with COVID-19 infection?
2. What is the relationship between the selected inflammatory biomarkers and fatigue among hospitalized patients with COVID-19 infection?

## 2. Literature Review

The current evidence suggested that the inflammatory responses play an important role in the progression of COVID-19. In a literature review study conducted by Rostami and Mansouritorghabeh [9], they found that the higher the levels of D-dimer, the worse the prognosis in patients with COVID-19 infection. An increase in the D-dimer levels by three or four times during the early stages of the disease was associated with an elevated level of death. The study concluded that the D-dimer test is a reliable predictor of identifying thrombosis in COVID-19 patients and disease prognosis [9].

Zeng et al. [8] conducted a literature review study using several databases with an aim to investigate the association between several inflammatory markers and the severity of COVID-19 disease. Sixteen studies were included in the analysis with around 4000 participants. The findings indicated that the patients with severe symptoms had significantly higher levels of procalcitonin (PCT), interleukin-6 (IL-6), ESR, and CRP than that of the nonsevere cases of COVID-19. However, these studies were heterogeneous and were conducted in a single country. In addition, these studies were reported to be underpowered indicating limited generalizability of these findings in exploring the mechanism of the effect of these biomarkers with the severity of COVID-19.

Even though fatigue was reported as a common symptom in COVID-19 infection [6], Poenaru et al. [10] reported a conflicting evidence. Poenaru et al. conducted a review study to investigate the etiology of fatigue in patients with COVID-19 infection. They stated that the inflammation per se might not be the source of fatigue. The review found several noteworthy similarities between COVID-19 symptoms and chronic fatigue syndrome in terms of symptom patterns, so, they concluded that there was no sufficient evidence to identify COVID-19 as a universal trigger for such symptoms.

## 3. Methods

### 3.1. Design

A cross-sectional design was used to assess the COVID-19 patients'fatigue and to determine the relationship with inflammatory biomarkers.

### 3.2. Sample

A convenience sampling strategy was used to recruit participants from the targeted hospitals. The inclusion criteria were the following: (1) patient who have confirmed diagnosis of moderate or severe COVID-19, (2) equal to or more than 18 years of age, and (3) can speak and read Arabic language. The exclusion criteria were those diagnosed as critical cases or intubated patients. The Jordanian Ministry of Health protocol [4] stated that the confirmed case of COVID-19 infection is defined as the case that is laboratory-confirmed by a PCR examination through a positive result to detect the SARS-CoV-2 virus.

The protocol stated that cases are diagnosed in Jordan only by adopting the polymerase chain reaction (PCR) test. Test samples are taken by using a nasopharyngeal swab, sputum sample, or a pulmonary lysing (BLM) sample. Two or more samples can be taken depending on the availability of the samples and according to the opinion of the attending physician, as required by the patient's condition.

COVID-19 infection in Jordan is diagnosed based on the results of the real-time PCR (RT-PCR) test (COVID-19 MDx RT-PCR COVID-19 detection kits). Those tests are FDA approved by the Jordanian Food and Drug Administration. The RT-PCR is an *in vitro* diagnostic technique that is used to detect SARS-CoV-2 by using a nasopharyngeal, oropharyngeal, mid-turbinate, or an anterior turbinate swap. Only trained staff are allowed to perform this test in predefined test locations. The reported clinical performance of these RT-PCR kits is 100% positive percent agreement, and 100% negative percent agreement by the manufacturer. Unfortunately, no reports were found regarding the test's sensitivity and specificity in Jordan. The estimated sensitivity of the test was reported around 80% and the specificity was about 99% [11].

The sample size was estimated using G*∗*Power 3.1 for multiple linear regression. The following parameters were used to estimate the sample size: alpha = 0.05, beta = 0.80, and effect size =  0.07 (representing small to medium effect size). The resulting estimation of the sample size was 267 participants. An estimated dropout percentage of 15% was considered. Thus, the minimum final estimated sample size was 307 participants (267 + 40), and the final recruited sample was 351 participants.

### 3.3. Instruments

#### 3.3.1. Fatigue

The Chalder fatigue scale was used to measure fatigue. This scale is composed of 11 items that talk about sensations and functionality. Each one of these items is measured using a 4-point Likert-type scale. The responses range from asymptomatic (no symptoms) to the maximum/highest symptomology. Responses on the far left receive a score of 0, and as the case becomes more symptomatic, the responses increase to 1, 2, or 3. The global fatigue score for each case was calculated by the summation of the scores, so that the global score can range from a minimum of 0 to a maximum of 33. In addition, the Chalder fatigue scale was used to determine the fatigue type, where items from 1 to 7 are used to measure the physical fatigue, and items from 8 to 11 measures the psychological fatigue. This scale can be used to score the values bimodally, i.e., if fatigue exists or not. In this case, items are scored as 0 or 1. A score of 4 or more using this bimodal measurement indicates the existence of fatigue (fatigue caseness). The questionnaire was translated into Arabic. The translation process was based on the recommendations by Sousa and Rojjanasrirat [12].

#### 3.3.2. Severity of COVID-19 Infection

The severity of COVID-19 infection was measured as an ordinal variable to determine whether the patient is considered to have a moderate or severe COVID-19 infection. The protocol was proposed by the diagnostic and treatment protocol for patients with the emerging coronavirus (COVID-19) issued by the Ministry of Health in Jordan and approved by the Jordanian National Committee for Epidemic Control; and it was used to describe if the case is considered to be moderate or severe COVID-19 infection as previously mentioned.

#### 3.3.3. Inflammatory Biomarkers

The results of the inflammatory biomarkers for the corresponding participants were gathered from the hospital records. The aforementioned protocol by the MOH described the required tests for any and all admitted/hospitalized patients with COVID-19 infection; and this protocol indicated that the following tests should be performed for the hospitalized COVID-19 patients on a daily basis.

The following are the inflammatory markers tested according to the management protocol:

- Neutrophils
- Lymphocytes
- Monocytes
- Eosinophil
- Basophil
- C-reactive protein (CRP)
- Procalcitonin (PCT)
- D-dimer
- Interleukin-6 (IL-6)
- Erythrocytes sedimentation ratio (ESR)

### 3.4. Settings

Three of the hospitals that were allocated by the Jordanian government as hospitals to receive patients with COVID-19 infection were selected for data collection. Only certain hospitals were selected by the government to admit COVID-19 patients. Other hospitals were instructed to transfer any COVID-19 patients to those previously identified as a precaution to control disease transmission.

### 3.5. Data Collection Procedure

IRB approval was received on April 20, 2022. Data collection was performed between May 1st, 2022, and June 4th, 2022. After acquiring the IRB and the approval from the selected hospitals, the investigator introduced the topic to the head nurse and staff nurses to provide clarification regarding the study and the questionnaire. The investigator distributed the questionnaire to the potential participants who matched the inclusion criteria. Those who accepted to participate signed an informed consent and completed the questionnaire. Results of the inflammatory markers were collected from the corresponding participant's records by the investigator.

### 3.6. Data Analysis Procedures

Data analysis was performed using the SPSS program. The variables that were tested in this study were reported using the descriptive statistics such as average, SD, frequency, range, and percentage. Pearson R, multiple regression analysis, chi-square test, and *t*-tests were used to answer the research questions. TheP value for this study was determined at the level of 0.05.

### 3.7. Ethical Consideration

The study method was approved by the ethical research committee and by the IRB of the Jordan University of Science and Technology (IRB number 656-2021; Jordan University of Science and Technology). A formal informed consent was obtained from the participants who decided to participate in the study. The data collection sheets and the questionnaires were coded so that the confidentiality of the participants is protected. The participation was completely and thoroughly voluntary, and the participants were assured that their answer sheets and their data will remain confidential. The participants were informed that they have the right to withdraw from the study at anytime during the conduction of the study, and that their withdrawal will be without any penalty. The participants were informed about the estimated time needed to complete the participation and to answer the questionnaire; and they received the contact information during the data collection. No harm or risk was imposed on the participants.

## 4. Results

### 4.1. Sociodemographic Characteristics

A total of 352 patients participated in the study. The average age was 57 years old (SD = 19 years), and more than half of the sample were female (*n* = 200). Not all participants reported having fatigue based on the Chalder scale. About a third of the sample (*n* = 130; 36.9%) reported no fatigue. Meanwhile, 63.1 percent of the sample reported having fatigue (*n* = 222). The overall fatigue score ranged within the study sample between 0 and 33 (mean = 16; SD = 8.3). Results regarding the levels of physiological and psychological fatigue showed that the range of physical and psychological fatigue were between 0 and 21, and 0 and 12, respectively. More than three-fourths of the patients had severe COVID-19 infection (*n* = 274, 77.8%), and the rest had moderate COVID-19 (*n* = 78, 22.2%) (See Table 1 for demographic characteristics).

### 4.2. COVID-19 Severity and Fatigue

The relationship between the severity of COVID-19 infection and fatigue was tested using Pearson R and showed that the severity of the infection had a significant positive correlation with the total fatigue score and physiological fatigue (*r* = 0.16, *p* < 0.05; *r* = 0.18, *p* < 0.05), respectively, but had no significant correlation with the psychological fatigue.

Further testing was performed using an independent sample *T*-test to assess the differences in fatigue level between the moderate and the severe COVID-19 infection groups. Both the total fatigue level and physiologic fatigue level were entered as test variables. The results supported the initial correlation tests and showed that there was a significant difference between the mean scores of fatigue in the two severity groups (moderate and severe) (*t* = −3.0, *p* < 0.05); and showed the presence of significant difference in the mean scores of physiological fatigue among these groups (*t* = −3.5, *p* < 0.05). In these two tests of difference, the mean score of fatigue was higher amongst the severe COVID-19 infection group than that of the moderate infection group. In addition, there were no significant differences between the mean scores of psychological fatigue and the mentioned severity groups. Additional testing was performed to further explore this relationship. A chi-square test was conducted between fatigue as bimodally (binary) and the severity of COVID-19 infection. The chi-square test showed that the severity of COVID-19 infection was independent from whether the participant had fatigue or not (chi-square = 1.24; *p* > 0.05).

### 4.3. Inflammatory Markers and Fatigue

To test the relationships between the selected inflammatory markers and level of fatigue, a multiple linear regression analysis was done. In this analysis, age, body temperature, and O_2_ saturation were entered with the previously identified inflammatory markers. The regression analysis showed that the overall model was significant (*F* = 6.3, *p* < 0.01) and the overall model accounted for about 20% of the variability of the fatigue scores (*R* square = 0.20; adjusted *R* square = 0.174).

However, only age, neutrophil count, and lymphocyte count were found to have a significant influence on the fatigue level (See Table 2). Age had a positive relationship with the total fatigue level (*B* = 0.151; *p* < 0.05) indicating that a difference of 10 years of age would increase the level of fatigue by approximately 1.5 points. Meanwhile, neutrophil and lymphocyte count had a negative (inverse) relationship with fatigue (*B* = 0.045; *B* = 0.082; *p* < 0.05 respectively), indicating that an increase in 100 units of neutrophil count would be associated with a decrease in the fatigue level by about 4.5 points, whereas 100 increments in lymphocyte would be associated with a decreased fatigue level by 5 points. Additional regression analyses were performed to assess if the relationships between the inflammatory markers and the type of fatigue (physical and psychological) were any different. The results remained the same and showed that only age, neutrophils, and lymphocytes had a significant influence on the level of physiological and psychological fatigue.

Further analysis was performed to examine the relationships between the inflammatory markers and fatigue. Binary logistic regression tests were performed to assess the ability of the level of inflammation (inflammatory markers) and to predict the fatigued and nonfatigued cases. In these tests, fatigue caseness were entered as binary independent factors and checked against the previously mentioned dependent factors. First, a multivariate binary logistic regression analysis was performed. The results of this test showed that the overall model was significant and was a better fit than the model with no predictors (model chi-square = 50.4, *p* < 0.05; Hosmer and Lemeshow test chi-square = 12.5, *p* > 0.05). In this model, only age and lymphocytes were significant predictors of fatigue caseness. The model proposes that an increase in age and a decrease in the lymphocyte count are associated with an increased likelihood of fatigue (See Table 3). The proposed model showed that the number of cases that were correctly predicted based on the observed value was 71.3%. However, the model had a high percentage of correctly predicting cases with fatigue (194 cases were correctly predicted to have fatigue versus 28 cases predicted to have fatigue but observed to not have fatigue), whereas 43.8% were correctly predicted by the model (57 correctly predicted vs. 73 not correctly predicted by the model to not have fatigue), indicating better model sensitivity compared to its specificity.

In the second analysis, a univariate binary logistic regression was conducted to assess if the results of the first test remained the same, or whether independent variables/factors' effects on fatigue differed (See Table 4 for the univariate logistic regression). Results showed that the findings remained about the same where only age and lymphocyte count significantly predicted fatigue caseness.

## 5. Discussion

This study aimed at examining the relationships between various inflammatory markers and fatigue in patients with COVID-19. Also, this study examined whether the severity of COVID-19 infection influences fatigue, and if the severity plays a role in the relationships between the level of the tested inflammatory markers and fatigue.

Studying fatigue during and post COVID-19 infection has been recognized as an important aspect of the research study due to the prevalence of this infection, the prevalence of fatigue in COVID-19 infection, and the long-term effect of fatigue on the quality of life, thus, fatigue ought to be screened and continued to be monitored in patients with COVID-19 infection [13] especially for that substantial proportion of people who got COVID-19 infection and continue to suffer from the ongoing fatigue or post viral fatigue [14].

This study showed that fatigue is a prominent symptom and reported a high prevalence of fatigue among patients with moderate and severe COVID-19 infection. Not many studies were found to address the prevalence of fatigue in hospitalized patients with moderate and severe COVID-19 infection. A study was found and reported results regarding the prevalence of fatigue that were congruent with our study. Shendy [15] reported about 64% prevalence of fatigue among their study sample (nonhospitalized mild and moderate cases of COVID-19 infection). Whereas the prevalence of fatigue in this study sample was about 63%.

Rudroff et al. [16] addressed fatigue in patients with COVID-19 infection and indicated that fatigue is one of the main symptoms of COVID-19 that negatively affect the patients and causes deterioration in their quality of life through decreasing patient's physical and psychological performance; and these deteriorations are attributed to the pathological changes caused by the disease process.

Our study found different results from that of Rudroff et al. [16] in that no direct relationship was found between most of the inflammatory markers and fatigue. This finding was substantiated in this study when this relationship remained the same even when assessed between inflammation and any of the two types of fatigue: the physical and psychological fatigue. These findings were congruent with commentary by Azzolino and Cesari [6] who stated that fatigue cannot be explained by a unique pathogenic mechanism. Such findings indicate that the relationship between inflammation and fatigue in patients with COVID-19 infection is complex and might be indirected or mediated by other factors. The results of the logistic regression further support this notion especially since lymphocyte count was the only inflammatory marker that contributed to the prediction of the presence of fatigue. Moreover, further investigation is required to describe the pathological processes that explain the rationale behind the inverse effect that lymphocyte count has on fatigue. Similar findings regarding the inverse relationship between lymphocyte and fatigue were reported by Illg et al. [17]; however, contradictory to our study this inverse relationship was mediated by the severity of COVID-19 infection.

Many studies in the literature also addressed the relationship between fatigue and the severity of COVID-19 infection such as Islam et al. [18] who reported in their review study that the severity of the infection as represented by the severity of the cytokine storm can influence the development of health problems such as fatigue. In addition, Poenaru et al. [10] in their review study reported that with regard to fatigue, multiple explanations regarding what influences fatigue exist, some suggested that COVID-19 patients experience dysregulations in their immune and neurological systems, and dysregulations in their metabolic pathways. However, these findings are not consistent with the other studies. The findings of our study were congruent with the reports of Islam et al. and Poenaru et al. where a significant relationship was found between the severity of COVID-19 infection and the total fatigue level. However, results showed that physiological fatigue was influenced by the severity of the infection and not psychological fatigue. Not only that, but also the severity of the infection played no role in the existence of fatigue, but the severity of the infection influenced its levels. These findings demonstrate the complexity of these relationships and the need to continue addressing fatigue among those with COVID-19 infection.

## 6. Conclusions

Fatigue in COVID-19 infection is prominent and is affected by many factors. Fatigue can be affected by the severity of the disease and the process of inflammation. However, many other factors may have a substantial influence on fatigue, and on the relationships between disease severity and process (inflammatory markers) on fatigue. The type of fatigue (physical, psychological) differed based on the severity of the disease. The severity of COVID-19 infection, disease process, levels of inflammatory markers, and fatigue are associated with each other in a complex relationship, and other factors may play a role in these effects. These findings necessitate the need to further test these relationships.

## 7. Limitations

The findings of the current study should be considered within the context of certain limitations. The participants were recruited using a nonprobability sampling technique (convenience sample). In addition, the authors collected the data from the participants at a single data point. Along the same line, fatigue was only measured during participants' hospitalization without performing a follow-up data collection to measure the level of fatigue after discharge. These limitations could limit the generalizability of the findings. Therefore, the authors recommend conducting future longitudinal studies with data collection from randomly selected participants to overcome these limitations.

## Figures and Tables

**Table 1:** Sociodemographic characteristics of the study sample.

	Mean	SD
Age	57	19
Total fatigue level	16	8.3
Physiological fatigue	10.6	5.5
Psychological fatigue	5.4	3.4
	Frequency (*n*)	Percentage (%)
Gender		
Male	152	43.3
Female	200	56.7
Marital status		
Single	56	15
Married	216	62
Divorced	52	15
Widow	28	8
Employment status		
Unemployed	207	58.8
Employed	90	25.6
Retired	55	15.6
Educational attainment		
Did not go to school	44	12.5
Elementary	27	7.7
High school/secondary	69	19.7
Diploma	97	27.4
Bachelor's degree/undergraduate	92	26.2
Postgraduate	23	6.6
Fatigue caseness		
No fatigue	130	39.1
With fatigue	222	63.1

**Table 2:** Multiple linear regression model to test the relationship between inflammatory markers and total fatigue levels.

Factor	*B*	*T*	*p*	95% CI lower bound	95% CI upper bound	Tolerance	VIF
Age	0.151	5.354	0.000^*∗*^	0.096	0.207	0.553	1.807
Temperature	−0.243	−0.461	0.645	−1.280	0.794	0.805	1.243
O_2_ saturation	−0.011	−0.226	0.821	−0.105	0.084	0.784	1.275
WBC	0.029	0.818	0.414	−0.041	0.100	0.734	1.363
Neutrophils	−0.045	−2.586	0.010^*∗*^	−0.080	−0.011	0.594	1.683
Lymphocytes	−0.082	−2.839	0.005^*∗*^	−0.138	−0.025	0.757	1.322
Monocytes	0.117	1.261	0.208	−0.066	0.300	0.683	1.465
Eosinophil	−0.029	−0.118	0.906	−0.521	0.462	0.553	1.809
Basophil	−2.506	−1.574	0.116	−5.638	0.625	0.861	1.161
CRP	0.003	0.641	0.522	−0.007	0.014	0.843	1.186
PCT	−0.054	−0.738	0.461	−0.196	0.089	0.907	1.102
D-dimer	−0.044	−0.163	0.871	−0.578	0.490	0.746	1.341
IL-6	0.008	0.999	0.318	−0.008	0.025	0.900	1.111
ESR	−0.021	−0.913	0.362	−0.067	0.025	0.693	1.443
COVID-19_severity	−0.409	−0.345	0.730	−2.741	1.923	0.683	1.464

^*∗*^Data with statistical significance (*p* < 0.05).

**Table 3:** Multivariate binary logistic regression with the fatigue caseness as a dependent factor.

Factor	*B*	SE	Wald	*d*f	*p*	OR	95% CI lower	95% CI upper
Age	0.031	0.008	21.301	1	0.000	1.040	1.023	1.057
Weight	−0.010	0.153	1.067	1	0.302	0.990	0.972	1.009
Temperature	0.066	0.018	0.184	1	0.668	1.069	0.789	1.447
O_2_ saturation	−0.015	0.015	0.998	1	0.318	0.986	0.958	1.014
WBC	0.015	0.011	1.856	1	0.173	1.015	0.993	1.038
Neutrophils	−0.006	0.005	1.542	1	0.214	0.994	0.984	1.004
Lymphocytes	−0.023	0.009	7.383	1	0.007	0.977	0.961	0.994
Monocytes	0.041	0.029	1.998	1	0.158	1.042	0.984	1.104
Eosinophil	0.027	0.073	0.138	1	0.710	1.027	0.891	1.184
Basophil	0.286	0.485	0.351	1	0.554	1.331	0.517	3.429
CRP	0.002	0.002	1.367	1	0.242	1.002	0.999	1.005
PCT	−0.007	0.021	0.111	1	0.740	0.993	0.954	1.034
D-dimer	−0.061	0.079	0.591	1	0.442	0.940	0.804	1.100
IL-6	0.003	0.003	1.638	1	0.201	1.003	0.998	1.008
ESR	−0.001	0.007	0.025	1	0.874	0.999	0.986	1.012
COVID-19_severity	−0.597	0.360	3.343	1	0.067	0.551	0.291	1.044
Constant	−2.212	6.268	0.144	1	0.704	0.109		

**Table 4:** Univariate logistic regression with the fatigue caseness as a dependent factor.

Factor	*B*	SE	Wald	*d*f	*p*	OR	95% CI lower	95% CI upper
Age	0.033	0.006	29.633	1	0.000	1.034	1.021	1.046
Weight	0.004	0.008	0.197	1	0.657	1.004	0.988	1.020
Temperature	−0.217	0.127	2.896	1	0.089	0.805	0.627	1.033
O_2_ saturation	−0.047	0.017	7.565	1	0.006	0.954	0.923	0.987
WBC	0.006	0.009	0.384	1	0.535	1.006	0.988	1.023
Neutrophils	−0.007	0.004	3.479	1	0.062	0.993	0.986	1.000
Lymphocytes	−0.013	0.007	3.942	1	0.047	0.987	0.974	1.000
Monocytes	0.048	0.022	4.697	1	0.030	1.050	1.005	1.097
Eosinophil	0.060	0.053	1.276	1	0.259	1.062	0.957	1.178
Basophil	0.228	0.416	0.302	1	0.583	1.256	0.556	2.836
CRP	0.003	0.002	3.538	1	0.060	1.003	1.000	1.006
PCT	−0.005	0.018	0.068	1	0.794	0.995	0.960	1.032
D-dimer	0.081	0.066	1.529	1	0.216	1.085	0.954	1.234
IL-6	0.001	0.002	0.291	1	0.590	1.001	0.997	1.006
ESR	0.005	0.005	0.724	1	0.395	1.005	0.994	1.015
COVID-19_severity	0.291	0.262	1.239	1	0.266	1.338	0.801	2.235

## Data Availability

The data used to support the findings of this study are available from the corresponding author upon request. Data are in the form of a SPSS file that the author has stored on his personal PC. Questionnaires were disposed of according to the institutional policy and regulation.

## Additional Points

*What does this study add?* (i) The evidence regarding the effect of the disease process in COVID-19 infection and the development of fatigue is still indistinct. (ii) The level and the type of fatigue were affected by the severity of the disease. (iii) Many other factors may have a substantial influence on fatigue, and on the relationships between disease severity and process (inflammatory markers) on fatigue.
